# A Simple and Efficient Method for the Substrate Identification of Amino Acid Decarboxylases

**DOI:** 10.3390/ijms232314551

**Published:** 2022-11-22

**Authors:** Mingyu Fang, Xing Wang, Zhikun Jia, Qiongju Qiu, Peng Li, Li Chen, Hui Yang

**Affiliations:** Shanghai Key Laboratory of Metabolic Remodeling and Health, Institute of Metabolism and Integrative Biology, Fudan University, Shanghai 200438, China

**Keywords:** amino acid decarboxylases, substrate identification, GAD65, AADC, enzymatic activity, LC-MS

## Abstract

Amino acid decarboxylases convert amino acids into different biogenic amines which regulate diverse biological processes. Therefore, identifying the substrates of amino acid decarboxylases is critical for investigating the function of the decarboxylases, especially for the new genes predicted to be amino acid decarboxylases. In the present work, we have established a simple and efficient method to identify the substrates and enzymatic activity of amino acid decarboxylases based on LC-MS methods. We chose GAD65 and AADC as models to validate our method. GAD65 and AADC were expressed in HEK 293T cells and purified through immunoprecipitation. The purified amino acid decarboxylases were subjected to enzymatic reaction with different substrate mixtures in vitro. LC-MS analysis of the reaction mixture identified depleted or accumulated metabolites, which corresponded to candidate enzyme substrates and products, respectively. Our method successfully identified the substrates and products of known amino acid decarboxylases. In summary, our method can efficiently identify the substrates and products of amino acid decarboxylases, which will facilitate future amino acid decarboxylase studies.

## 1. Introduction

Amino acid decarboxylation is a common and important biological process in amino acid catabolism of animals, plants, and microorganisms [1]. Amino acid decarboxylases catalyze the conversion of amino acids to CO_2_ and corresponding biogenic amines, which are involved in the synthesis of neurotransmitters and other important small molecules. For example, ornithine decarboxylase (ODC) converts L-ornithine into putrescine, which is the precursor of spermidine and spermine. Spermidine, spermine, and putrescine, collectively known as polyamines, are important for the growth, development, and repair of tissues. Increased levels of ornithine decarboxylase and polyamines have been reported in various cancers [2]. S-adenosylmethionine decarboxylase (SAMDC) catalyzes the decarboxylation of S-adenosylmethionine to provide propyl amino groups for the conversion of putrescine into spermidine and spermine, which regulate tumorigenesis and the proliferation of tumor cells [3].

**Figure 1:**
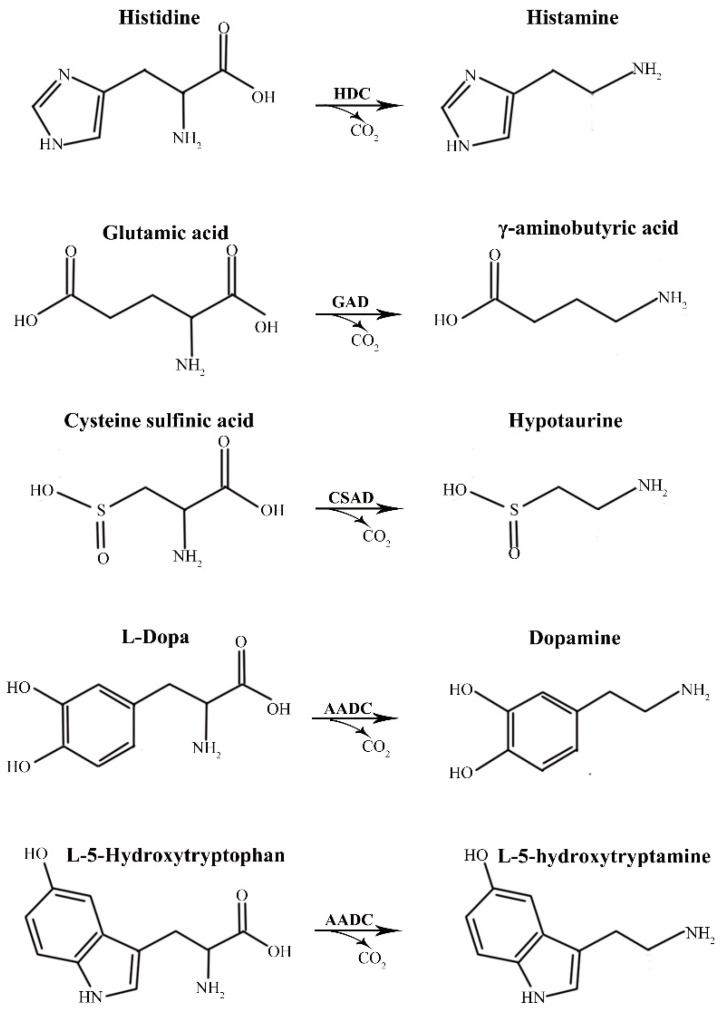
Substrates and products of human group II amino acid decarboxylases. HDC, histidine decarboxylase; GAD, glutamic acid decarboxylase; CSAD, cysteine sulfinic acid decarboxylase; AADC, aromatic amino acid decarboxylase.

In eukaryotes, there is a group II amino acid decarboxylase superfamily that catalyzes the synthesis of different biogenic amines, including glutamic acid decarboxylase (GAD), aromatic amino acid decarboxylase (AADC), histidine decarboxylase (HDC), and cysteine sulfinic acid decarboxylase (CSAD) (Figure 1). GAD catalyzes L-glutamate to γ-aminobutyric acid (GABA), an important inhibitory neurotransmitter [4,5]. GABA is associated with many neurological diseases, such as stiff person syndrome [6], schizophrenia [7] and Huntington’s disease [8]. GAD consists of two subtypes, GAD65 and GAD67, which are encoded by different genes [9]. AADC catalyzes levodopa (L-Dopa) and L-5-hydroxytryptophan to produce dopamine and serotonin, respectively, both of which are bioactive neurotransmitters. Dopamine is related to a variety of neurological diseases, such as Parkinson’s syndrome [10] and schizophrenia [11]. Serotonin is abundantly present in the digestive system and is closely related to the movement and secretion of the gastrointestinal tract, animal emotion regulation, learning, and memory [12]. HDC catalyzes histidine to histamine, which is involved in different physiological processes, including nerve signaling [13], cell proliferation [14], gastric acid secretion [15,16], and immune responses to allergies and inflammation [5,17]. CSAD catalyzes cysteine sulfinic acid to hypotaurine, which is further oxidized to taurine. Taurine is a nutritional factor for the development of the central nervous system [18], and plays an important role in the cardiovascular system [19], the hepatobiliary system [20], and the treatment of diabetes [21].

Since amino acid decarboxylases and their products are involved in many biological processes and are closely related to many diseases, it is of great biological significance to identify the corresponding substrates and products of amino acid decarboxylases. Over the past few decades, several methods have been used to detect the enzymatic activity and products of the amino acid decarboxylases, such as HPLC [22], radiochemical methods [23], and spectrophotometry [24]. With the rapid development of DNA sequencing technology, new genes with similarities in sequence or structure to amino acid decarboxylase have been discovered. However, the function of these candidate decarboxylases remains unknown. Identification of the substrate and product of a decarboxylase will be the first and foremost step to understand its biological function.

To this end, we established a method for the simple and accurate identification of the enzymatic activities and substrates of amino acid decarboxylases based on LC-MS. We enriched and purified amino acid decarboxylases (GAD65 and AADC) in vitro, and performed reactions with substrate mixtures of different sources (amino acid mixture, 1640 medium, and metabolites of mouse tissues), and then monitored depleted or accumulated metabolites, which would be the enzyme substrates and products, respectively. Our method successfully identified the enzyme activities of several known amino acid decarboxylases in vitro, which validates its future application for studying other unknown amino acid decarboxylases.

## 2. Results

### 2.1. Experimental Procedure for the Substrate Identification of Amino Acid Decarboxylases

**Figure 2:**
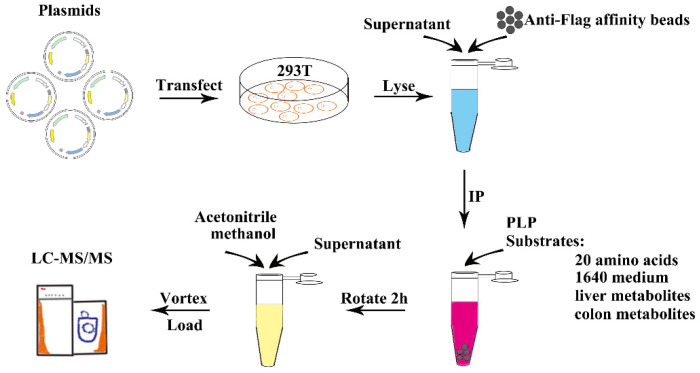
Workflow for the substrate identification of amino acid decarboxylase. IP, immunoprecipitation; PLP, Pyridoxal Phosphate.

In this study, we used GAD65 and AADC to demonstrate our method for the substrate identification of amino acid decarboxylases. The workflow consisted of 4 steps: (1) Expression and purification of the amino acid decarboxylase. We cloned the cDNAs of the two genes, GAD65 and AADC, into the eukaryotic expression vector pcDNA3.1, and added a FLAG tag peptide to the N-terminus of the protein for fusion expression. The recombinant plasmids with the FLAG-tagged peptide were transiently transfected into HEK 293T cells. After 36 h, the cells were lysed, and the supernatant of the cell lysate was centrifuged to collect and add with anti-FLAG agarose beads. GAD65 and AADC fused with FLAG tag were enriched by immunoprecipitation; (2) The enzymatic reaction of the amino acid decarboxylase. The enzyme cofactor PLP, substrate mixtures (20 amino acid mixture, 1640 medium, or metabolites from mouse liver and colon) were incubated with the agarose beads enriched with GAD65 and AADC for 2 h at room temperature; (3) Extraction and detection of metabolites. After the reaction, the products were extracted with acetonitrile: methanol (1:1), and the mixture after the reaction was detected by LC-MS; (4) Data analysis. We compared metabolite abundance between the vector control group and the decarboxylase group. Metabolite depleted in the decarboxylase group was the possible enzyme substrate, while metabolite accumulated was the possible product (Figure 2).

### 2.2. Purification of GAD65 and AADC

**Figure 3:**
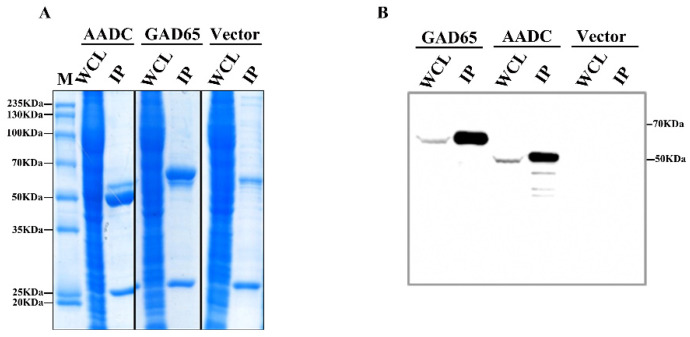
Expression and enrichment of GAD65 and AADC. (**A**) Plasmids Vector, FLAG-AADC, and FLAG-GAD65 were each transfected into HEK 293T cells. After 36 h, the cells were collected, and the whole cell lysate (WCL) after centrifugation was immunoprecipitated with anti-FLAG beads. IP, immunoprecipitation. The WCL and IP samples were subjected to SDS–PAGE gel electrophoresis and stained. Lane M served as a protein marker. (**B**) The WCL and IP samples were detected by Western blotting, and the expression of the target proteins was detected by anti-FLAG antibody.

To obtain high-purity amino acid decarboxylases for enzymatic reactions, we firstly purified GAD65 and AADC in vitro. The fusion expression recombinant plasmids FLAG–AADC and FLAG–GAD65 were transiently transfected into HEK 293T cells, and the empty vector (Vector) was transfected as a negative control. The cell lysates were collected and incubated with anti-FLAG beads to enrich the GAD65 and AADC, and a small amount of the enriched proteins were taken for SDS–PAGE electrophoresis and Coomassie brilliant blue analysis (Figure 3A). GAD65 and AADC were significantly enriched in the samples compared with the vector after immunoprecipitation. To further verify the enriched proteins, the result of Western blotting showed that the two decarboxylases GAD65 and AADC were highly enriched (Figure 3B).

### 2.3. Detecting the Enzymatic Activities of GAD65 and AADC in a Simple System

**Figure 4:**
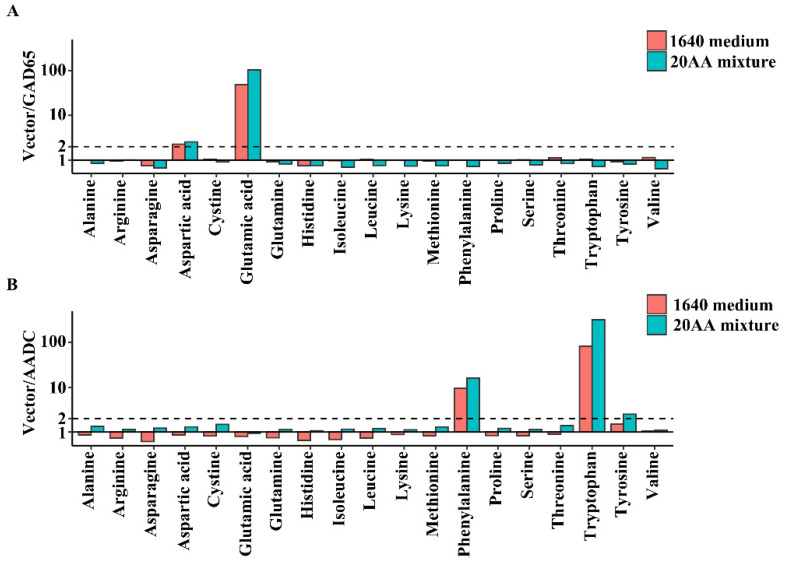
Detection and analysis of enzymatic activity reactions of GAD65, AADC with 20 amino acid mixture or 1640 medium. (**A**) The purified Vector and GAD65 were subjected to enzymatic reaction with 20 amino acids or 1640 medium, respectively. After incubation at room temperature for 2 h, the samples after enzymatic reaction were extracted and analyzed by LC-MS. (**B**) The purified Vector and AADC were subjected to enzymatic reaction with 20 amino acids or 1640 medium, respectively. After incubation at room temperature for 2 h, the samples after enzymatic reaction were extracted and analyzed by LC-MS.

To detect the enzymatic activities of the decarboxylases that we purified, we performed an in vitro enzymatic reaction with a mixture of 20 amino acids or 1640 medium. In this simple system, GAD65 significantly reduced glutamate signal compared to the Vector group (Figure 4A), which indicated that the purified GAD65 catalyzed glutamate decarboxylation specifically. The incubation of the purified AADC with the mixture of 20 amino acids or 1640 medium showed that phenylalanine, tyrosine, and tryptophan were reduced, which indicated that the purified AADC had catalyzed aromatic amino acid decarboxylation. It was worth noting that the phenylalanine and tryptophan signals decreased more dramatically than the tyrosine after the addition of AADC, which may indicate that AADC had stronger substrate recognition ability and catalytic activity for phenylalanine and tryptophan (Figure 4B). In conclusion, our method not only showed substrate specificity, but also revealed substrate preference for both GAD65 and AADC, which validated the specificity and sensitivity of our method.

### 2.4. Detecting the Substrate and Enzymatic Activity of GAD65 in a Complex System

When detecting the enzymatic activity of a decarboxylase with unknown function, its substrate may not be the common amino acid used in the above reaction system. We developed a high-throughput untargeted metabolomics method to test whether we could identify the substrate in a more complex substrate reaction system.

**Figure 5:**
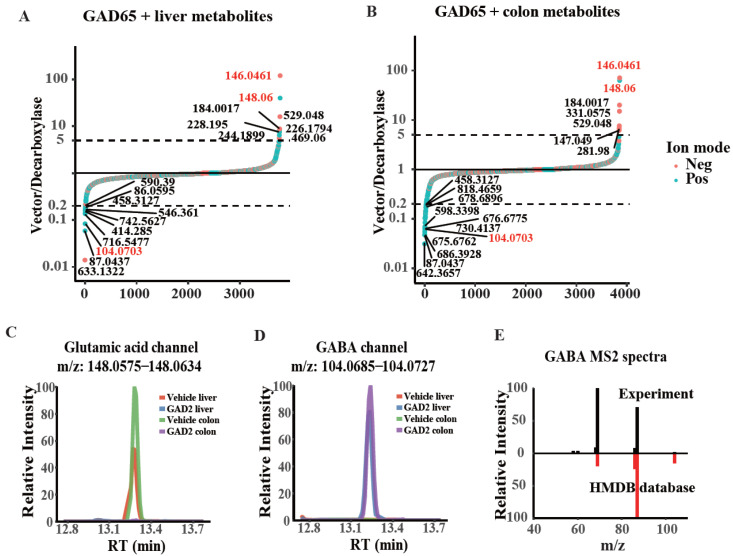
Detection and analysis of the substrate and product of GAD65 with tissue metabolites. (**A**,**B**) LC-MS untargeted metabolomic results for enriched Vector and GAD65 subjected to enzymatic reaction with liver metabolites (**A**) and colonic metabolites (**B**). The signal ratio between the vector and decarboxylase sample was plotted. Metabolite depleted in the decarboxylase sample showed a higher vector/decarboxylase ratio. (**C**,**D**) Ion chromatograms of glutamate (**C**) and GABA (**D**). (**E**) Experiment and database MS2 spectra confirmed the new peak was GABA.

We extracted the metabolites of mouse liver and colon tissue and incubated the metabolites with the enriched GAD65. We calculated the signal ratio between the vector and GAD65 for each metabolite, and ranked all from low to high (Figure 5A,B). In both liver and colonic metabolites samples, the glutamate signal in both negative mode (*m*/*z* = 146.0461) and positive mode (*m*/*z* = 148.0600) stood out among all measured metabolites (Figure 5A,B). Examining the chromatographic peaks in these samples confirmed glutamate depletion upon adding GAD65 (Figure 5C). Meanwhile, a metabolite corresponding to glutamate decarboxylation (*m*/*z* = 104.0703) showed strong accumulation in both liver and colonic metabolites samples (Figure 5A–C). MS/MS experiments confirmed the new peak was GABA, which is the known product of GAD 65 (Figure 5E). In conclusion, our method identified both the substrate and product of GAD65 in the complex substrate reaction system, which provides the basis for the enzymatic activity detection and substrate identification of other decarboxylases with unknown functions. 

## 3. Discussion

Our present work provides a simple and efficient method for the identification of the substrate and product of an amino acid decarboxylase. Through the eukaryotic protein expression system, immunoprecipitation protein purification, and in vitro enzymatic reaction with complex metabolite mixtures and LC-MS detection, we validated our method by identifying both the substrate and product for the amino acid decarboxylase GAD65. Combining the eukaryotic expression system and the immunoprecipitation protein purification allows for the method to be adapted to various proteins of interest. Using the tissue extract to serve as a metabolite pool in the in vitro enzymatic reaction was the highlight of our method. Instead of testing metabolites one by one, the tissue extract contained more diverse metabolites, which significantly increases the scope of tested metabolites. It may be more important to study decarboxylases with tissue-specific expression, as the corresponding tissue extract is more likely to contain the enzyme’s substrates. In addition, we observed that metabolites similar to the known substrates of the enzyme showed partial depletion, e.g., aspartate in the GAD65 reaction (Figure 4), which suggested that our method is suitable to identify other potential substrates with the presence of the main substrate. By leveraging the high resolution and sensitivity of LC-MS, our method has high efficiency and specificity to identify changing metabolites in the complex system. In conclusion, our method is poised to identify substrates and products for other novel amino acid decarboxylases, which will shed light on the understanding of their biological functions.

## 4. Materials and Methods

### 4.1. Materials

Eight-week-old male C57BL/6J mice were purchased from Gempharmatech, the high-glucose medium was purchased from MACGENE (Beijing, China) (Cat. No.: CM10013), and anti-DYKDDDDK (FLAG) affinity beads were purchased from Changzhou Smart-Lifesciences (Cat. No. SA042005). Transfection reagent polyJet was purchased from SignaGen Laboratories (Cat. No. SL100688), protease inhibitor cocktail was purchased from Roche (Cat. No. 4693159001), the 20 amino acid mixture was purchased from Sigma, and 1640 medium was purchased from Gibco (Cat. No. 22400071). Pyridoxal phosphate (PLP, sigma, Cat. No. P9255), acetonitrile (HPLC grade, Thermofisher, Waltham, MA, United States, A998-4), and methanol (HPLC grade, Thermofisher, Waltham, MA, United States, A452-4) were used in this study.

### 4.2. Extraction of Tissue Metabolites for Enzymatic Assay

For mouse liver samples, 1 g of mouse liver tissue was weighed and added to 750 μL of sterile water, then homogenized with high-throughput grinder with a cycle of 15 s homogenization at 60 Hz frequency and 5 s rest for four repetitions. For mouse colon samples, longitudinally sectioned colon was washed three times with saline to ensure that the colon contents were completely rinsed. The proximal colon tissue was about 1.5 cm long, weighed and placed in a 2 mL EP tube, and then added to 1 mL of sterile water. After tissue grinding, both liver and colon samples were subjected to ultrasonic lysis at 100W for 3 s and then stopped for 3 s, for a total of 1 min. Then the samples were heated at 95 °C for 10 min and centrifuged at 22,000× *g* for 10 min at 4 °C. The supernatant was aliquoted at 100 μL/tube and stored at −80 °C.

### 4.3. Protein Expression and Purification

The human cDNAs of GAD65 and AADC were constructed into a pcDNA3.1 vector, and a FLAG tag (GACTACAAAGACGATGACGATAAA) was fused to the N-terminus of the cDNAs of GAD65 and AADC. Their respective plasmids were transfected into HEK 293T cells. The day before transfection, the cells were plated onto a 10 cm dish at a density of about 50% of the confluence rate, and the cells were transfected the next day when the confluence rate was about 70%-80%. Each 10 cm dish was transfected with 10 μg of plasmid, and the medium was changed 4-6 h after transfection. Cells were harvested 36 h after transfection. Cell pellets were resuspended in 1 mL of RIPA buffer (50 mM Tris, 150 mM NaCl, 1% Triton X-100, 1 mM EDTA, pH 7.4). A protease inhibitor cocktail was added to the RIPA buffer before use. The cells were fully lysed on ice for 30 min and centrifuged at 12,000 rpm for 15 min at 4 °C. The lysate supernatant after centrifugation was transferred into an EP tube containing 50 μL of anti-FLAG affinity beads and incubated with a rotator at 4 °C for 4 h. Then the beads were centrifuged at 2000 rpm for 5 min to remove the supernatant and washed five times in RIPA buffer to obtain the purified proteins.

### 4.4. Enzymatic Activity Assay of GAD2 and AADC with Substrates from Different Sources

The stock solution of 20 amino acids was prepared with 20 mM ammonium acetate at a concentration of 10 mM, and the stock solution of PLP was at a concentration of 5 mM. The 20 amino acids were mixed and the reaction concentration of the amino acids was 100 μM. PLP was added to the amino acid mixture, 1640 medium, or cellular and tissue metabolites to make the reaction concentration of 100 μM. We divided the beads enriched for GAD65 or AADC into four equal parts, and added 100 μL of the mixture of amino acids, 1640 medium, or tissue metabolites, respectively; then placed them on a rotator at room temperature for 2 h.

### 4.5. Extraction of Metabolites for LC-MS Analysis

After the reaction, 100 μL of supernatant was added to a centrifuge tube containing 400 μL of 1:1 acetonitrile: methanol (pre-cooled at −20 °C); after brief vertexing, the samples were placed at −80 °C for 2 h to fully precipitate the protein. The extraction mixture was centrifuged at 4 °C for 10 min, and the supernatant was transferred to an LC-MS sample vial for mass spectrometry detection.

### 4.6. Targeted LC-MS Measurement

Targeted LC-MS/MS analysis was performed using a Shimadzu LC system coupled to a triple quadrupole mass spectrometer (QTRAP 6500+, ABSciex). A HILIC column (Waters XBridge^®^ Amide, 3.5 μm, 4.6 × 100 mm) was used for metabolite separation. Liquid chromatography was performed with 20 mM of ammonium acetate and 0.1% ammonium hydroxide in 95:5 water/ACN as phase A, and ACN as phase B. The flow rate was 0.4 mL/min, the total run time was 26 min, and the gradient was: 0 min, 85% B; 0.1 min, 85% B; 3.5 min, 32% B; 12 min, 2% B; 16.5 min, 2% B; 17 min, 85% B; 25.5 min, 85% B; 26 min, 85% B. ESI parameters setup: GS1, 33; GS2, 33; CUR, 25; temperature, 475; ISVF, 4500 or −4500 in positive or negative modes. The transitions for amino acids were: alanine, Q1 = 90.1, Q3 = 44.2, CE = 13 V; arginine, Q1 = 175.02, Q3 = 60, CE = 16V; asparagine, Q1 = 133.1, Q3 = 74, CE = 19V; aspartic acid, Q1 = 134, Q3 = 74, CE = 17V; cystine, Q1 = 241.002, Q3 = 74, CE = 32V; glutamic acid, Q1 = 148.1, Q3 = 84.1, CE = 17V; glutamine, Q1 = 147.1, Q3 = 84.1, CE = 17V; histidine, Q1 = 156.1, Q3 = 110.1, CE = 14V; isoleucine, Q1 = 132.1, Q3 = 86, CE = 13V; leucine, Q1 = 132.1, Q3 = 86, CE = 13V; lysine, Q1 = 147, Q3 = 67, CE = 32V; methionine, Q1 = 150.1, Q3 = 133, CE = 12V; phenylalanine, Q1 = 166.1, Q3 = 103, CE = 30V; proline, Q1 = 116.1, Q3 = 70.1, CE = 13V; serine, Q1 = 106, Q3 = 60, CE = 15V; threonine, Q1 = 120, Q3 = 74, CE = 13V; tryptophan, Q1 = 205, Q3 = 146, CE = 18V; tyrosine, Q1 = 182.1, Q3 = 77, CE = 39V; valine, Q1 = 118.1, Q3 = 55.2, CE = 13V. The injection volume for each run was 5 μL.

### 4.7. Untargeted LC-MS Measurement

Untargeted LC-MS/MS analysis was performed using a Shimadzu LC system coupled to a TripleTOF mass spectrometer (QTOF 6600+, ABSciex, made in Woodlands Central Industrial Estate, Singapore). A HILIC column (Waters XBridge^®^ Amide, 3.5 μm, 4.6 × 100 mm, made in Ireland) was used for metabolite separation. Liquid chromatography was performed with 20 mM of ammonium acetate, 0.1% ammonium hydroxide, and 5 μM ammonium phosphate in 95:5 water/ACN as phase A, and ACN as phase B. The flow rate was 0.4 mL/min, total run time was 21.6 min, and the gradient was: 0 min, 85% B; 2 min, 85% B; 3 min, 75% B; 7 min, 75% B; 8 min, 70% B; 9 min, 70% B; 10 min, 50% B; 12 min, 50% B; 12.1 min, 5% B; 15.5 min, 5% B; 15.6 min, 85% B; 21.6 min, 85% B. ESI parameters setup: GS1, 60; GS2, 60; CUR, 40; temperature, 500; ISVF, 5500 or −4500 in positive or negative modes. The injection volume for each run was 5 μL.

### 4.8. LC-MS Data Analysis

Targeted LC-MS/MS files were first converted to the mzML format using MSConvert software (ProteoWizard, version 3_0_11392). The mzML files were then processed by EL-MAVEN software (v0.12.0) to generate a peak table. Untargeted LC-MS/MS files were first converted to the mzXML format using MSConvert. The mzXML files were imported into MZmine [25] to perform mass detection, ADAP chromatogram building [26], smoothing, local minimum feature resolving, join aligning, gap filling, and duplicate feature filtering sequentially. The parameters were as follows: mass detection noise level, 100.0; *m*/*z* tolerance, 0.002 *m*/*z* or 20.0 ppm; chromatogram building group intensity threshold, 200.0; min group size in the number of scans, 5; smoothing algorithm, Savitzky–Golay; feature resolver chromatographic threshold, 0.85; min ratio of peak top/edge, 1.7; min number of data points, 5; join aligner retention time tolerance, 0.2 min; weight for *m*/*z* or RT, 1; gap filling intensity tolerance, 1.0; minimum data points, 1. Metabolite identification was achieved by comparing RT to the in-house standard and matching experimental MS2 to the HMDB MS2 library.

## Data Availability

The data is contained within the article.
